# Isolation and characterization of phthalates from Brevibacterium mcbrellneri that cause cytotoxicity and cell cycle arrest

**DOI:** 10.17179/excli2017-145

**Published:** 2017-03-24

**Authors:** Maheshwari Rajamanikyam, Varahalarao Vadlapudi, Sai Prathima Parvathaneni, Dhevendar Koude, Prabhakar Sripadi, Sunil Misra, Ramars Amanchy, Suryanarayana Murty Upadhyayula

**Affiliations:** 1 Biology Division, CSIR-Indian Institute of Chemical Technology (IICT), Uppal Road, Tarnaka, Hyderabad -500 007, India; 2 Crop Protection Chemicals Division, CSIR-Indian Institute of Chemical Technology, Uppal Road, Tarnaka, Hyderabad -500 007, India; 3 National Centre for Mass Spectrometry, CSIR-Indian Institute of Chemical Technology, Uppal Road, Tarnaka, Hyderabad -500 007, India; 4 NIPER Guwahati, 1st Floor, Institute of Pharmacy, Guwahati Medical College & Hospital Guwahati -781 032, India

**Keywords:** Brevibacterium mcbrellneri, di-(2-ethylhexyl) phthalate, dibutyl phthalate, anti-bacterial, Mosquito larvicidal, cytotoxicity, cell cycle arrest

## Abstract

Bacteria belonging to the family Brevibacterieae are ubiquitous Gram positive organisms that are responsible for the feet odour and cheese aroma. *Brevibacterium mcbrellneri* is a relatively new member belonging to Brevibacterieae. In the current manuscript we discuss isolation of biologically active metabolites from *Brevibacterium mcbrellneri*. Two aromatic esters were isolated from *Brevibacterium mcbrellneri* by “Bioassay guided fractionation strategy” and identified as di-(2-ethylhexyl) phthalate and dibutyl phthalate by chemical characterization using biophysical techniques. The phthalate compounds show broad spectrum antibacterial activity and mosquito larvicidal activity. Mosquito larvicidal activity has been attributed to inhibition of acetylcholinesterase enzyme activity. These compounds were found to be cytotoxic in multiple cell lines causing cell cycle arrest in G1 phase.

## Introduction

*Brevibacterium mcbrellnerii* is the member of genus *Brevibacterium *of *Brevibacteriaceae *family (Roux and Raoult, 2009[29]). *B. mcbrellnerii *is aerobic, non sporulating, Gram positive rod to coccoid shaped and grows in saline environment. This distinct species was initially isolated from clinical sample (McBride et al., 1993[22]), but can also be isolated from soil. A few biologically active metabolites have been isolated from this genus viz., 1, 6-phenazinediol 5, 10-dioxide (Iodinin) (Podojil and Gerber, 1967[27]), 6-Hydroxymethyl-1-phenazine-carboxamide and 1,6-phenazinedimethanol showing (Choi et al., 2009[5]), polyhydroxy butyrate (PHB) (Kiran et al., 2014[15]). The aim of our present study was to isolate, characterise and determine toxic compounds from *Brevibacterium mcbrellneri*. 

Two aromatic ester with anti-bacterial, larvicidal and cytotoxic properties di-(2- ethylhexyl) phthalate (DEHP) and dibutyl phthalate (DBP), were isolated whose chemical structures were determined through analysis of NMR and ESI-HRMS data. These compounds were isolated for the first time from this source. In recent years, phthalates have been isolated from strains of bacteria and fungi collected from diverse environments such as water, soils, plants and sediments. The most common fatty foods such as milk, butter, and meat were reported to have DEHP and other phthalates as contaminants, with a special mention about its toxicity at higher concentrations (Heinemeyer et al., 2013[12]). Both DEHP and DBP have been previously reported as secondary metabolite produced from various sources like brown algae, *Undaria pinnatifida* and *Laminaria japonica*, green alga, *Ulva* sp., red algae *Bangia atropurpurea* (Chen, 2004[3]). DEHP has been individually isolated from *Streptomyces bangladeshensis* (Al-Bari et al., 2005[1]), *Penicillium olsonii* (Amade et al., 1994[2]), *Alchornea cordifolia* (Mavar-Manga et al., 2008[21]), *Aloe vera* (Lee et al., 2000[18]), *Euphorbia cyparissias* and *Euphorbia seguieriana* (Toth-Soma et al., 1993[33]) etc., while DBP has been reported from *Streptomyces nasri*, *Streptomyces melanosporofaciens* (Lee, 2000[17]), *Streptomyces albidoflavus* (Roy et al., 2006[30]). In our present study both DEHP and DBP were found to have potent antibacterial activity, mosquito larvicidal activity due to acetylcholinesterase inhibitory activity and cytotoxicity due to cell cycle arrest in G1 phase. 

## Materials and Methods

### Chemicals and reagents

Nutrient Broth (Cat # M002, Himedia, India), Nutrient agar (Cat # M001, Himedi, India), Tryptone Soya Broth (Cat # LQ508, Himedia, India), Tryptone Soya Agar (Cat # M593, Himedia, India), Potato dextrose broth (Cat # M403, Himedia, India), Potato dextrose agar (PDA) (Cat # M096, Himedia, India), Osmium tetroxide (Cat # O5500, Sigma-Aldrich, USA), Fetal bovine serum (FBS) (Cat # 10270106, Thermo Fisher, USA), Dulbecco's modified Eagle's medium (Cat # D5648, Sigma-Aldrich, USA), Sodium pyruvate (Cat # S8636, Sigma-Aldrich, USA), Glutaraldehyde (Cat # G7776, Sigma-Aldrich, USA), 2, 2-dimethoxypropane (Cat # D136808, Sigma-Aldrich, USA), Ethyl acetate (Cat # 38311, SDFCL. India), n-Hexane (Cat # 20387, SDFCL. India), Silica gel (Cat # 109385, Merck, India), Potassium bromide (Cat # P0838, Sigma-Aldrich, USA), DTNB (Cat # D8130, Sigma-Aldrich, USA), Acetylthiocholine iodide (Cat # A5751 , Sigma-Aldrich, USA), CDCl3 (Cat # 570699, Sigma-Aldrich, USA), Isopropanol (Cat # 190764, Sigma-Aldrich, USA), Propidium iodide (Cat # P4170, Sigma-Aldrich, USA), Doxorubicin (Cat # D1515, Sigma-Aldrich, USA), Triton-X 100 (Cat # T8787, Sigma-Aldrich, USA), RNase A (Cat # R6148, Sigma-Aldrich, USA). 

### Microbial cultures and maintenance 

#### Bacterial cultures 

The antibacterial evaluation was carried out against Gram positive bacteria; *Staphylococcus epidermidis* (MTCC 435), *Bacillus subtilis* (MTCC 441), *Staphylococcus aureus *(MTCC 96), and Gram negative bacteria; *Pseudomonas aeruginosa* (MTCC 741)*, E. coli* (MTCC 443) and *Klebsiella pneumoniae* (MTCC 618). *Brevibacterium mcbrellneri* MTCC (3913) was chosen for isolation of biologically active compounds. 

#### Fungal cultures 

The antifungal evaluation was carried out against *Saccharomyces cervisiae *(MTCC 36), *Candida albicans *(MTCC 227), *Aspergillus flavus *(MTCC 277)*,* and* Aspergillus niger *(MTCC 282) (Xie et al., 2016[34]).

All the bacterial and fungal cultures were procured from Microbial Type Culture Collection, Institute of Microbial Technology (MTCC, IMTECH) Chandigarh, India. The bacterial stock cultures were maintained on nutrient broth and agar and fungal cultures on potato dextrose broth and agar. Sub culturing was done at regular intervals and stored at 4 °C (Holt, 1975[13]).

#### Mosquito larval cultures and maintenance 

The early 4^th^ instar larvae of *Aedes aegypti *were selected for evaluation of mosquito larvicidal activity. The larvae were collected and fed with a mixture powdered yeast and dog biscuits. These were maintained in insectaries till the emergence of adults. The colony was photo periodically maintained at 14 L: 10 D (hours) and with relative humidity 80 ± 5 % at 28 °C (Kumar et al., 2010[16]). Adult mosquitoes were fed with blood for egg maturation. The eggs were allowed to hatch in an enamel bowl lined with Whatman filter paper filled with distilled water. The pupae formed were separated and transferred to insectary for the emergence of adults (da Silva et al., 2016[6]).

#### Mammalian cell culture

The MTT cell proliferation was assessed against the cell lines HeLa (ATCC; CCL-2), A549 (ATCC; CCL-185), MCF-7 (ATCC; HTB-22), DU145 (ATCC; HTB-81), CHO (ATCC; CCL-61), HEK293 (ATCC; CRL-1573), NIH3T3 (ATCC; CRL-1658) were procured from the American Type Culture Collection (ATCC). These cell lines were grown and maintained in Dulbecco's modified Eagle's medium with non-essential amino acids, 10 % fetal bovine serum (FBS), 1 mM sodium pyruvate, in humidified atmosphere containing 5 % CO_2 _at 37 °C (Esumi et al., 2004[7]).

#### Preparation of culture for microscopy 

*Brevibacterium mcbrellneri* was Gram stained and observed under light microscopy at 100x resolution. For scanning electron microscopy the cells were collected by centrifugation, fixed with 2.5 % glutaraldehyde in 100 mM cacodylate buffer (pH 7.4) for 2 hrs at room temperature and post fixed for 2 hrs in 1 % osmium tetroxide. Cells were then washed twice with cacodylate buffer and retained by filtration in Millipore filters (0-20 mm diameter). After giving ethanol wash, filters containing the cells were dried (Haggis and Phipps-Todd, 1977[11]), covered with cover slip and finally coated with gold, giving a layer 40 nm thick. The cells were observed with a scanning electron microscope Model 3400N SEM (Hitachi, Japan).

#### Bacterial culture for extraction of secondary metabolites 

The well grown single colonies of *Brevibacterium mcbrellneri* were inoculated in subculture agar slant containing trypotone soya agar medium (gm/Lt): pancreatic digest of casein 15.0; enzymatic digest of soya bean 5.0; sodium chloride 5.0; agar 15.0; at pH 7 for 7 days at 37 °C. The obtained grown agar slant was served to inoculate 500 ml Erlenmeyer flask, each containing 100 ml of the medium. The culture medium was cultivated in the static position for 7 days. After harvesting, the resultant biomass, including the medium, was centrifuged at 10,000 rpm for 20 min at 4 °C. The biomass was separated and the remaining filtrate was extracted using ethyl acetate (1:1 v/v), and collected aqueous ethyl acetate was concentrated in-vacuo in rota-evaporator at 45 °C to afford 9.0 g as brown oily crude extract (Futamura et al., 2001[8]). The obtained unique brown organic extract was applied for further biological screening. According to the TLC monitoring of the crude extract solvent system for further purification was identified. 

#### Purification of biologically active compounds 

Isolation of active compounds was carried out using solvent extraction method (Chen and Huang, 2016[4]). For isolation of active toxic compounds, the crude extract was chromatographed in a column packed (2.5 i.d. x 50 cm) with silica gel (60-120 mesh) as stationary phase and hexane: ethyl acetate as mobile phase. The polarity was gradually increased to elute the mixture of compounds present in crude extract. The eluted fractions were TLC monitored and the biologically active fractions were further chromatographed to obtain two compounds. The compound isolated, from active subfractions were identified by chemical characterization on the basis of ^1^H NMR and ^13^C NMR, FT-IR, ESI-MS, and HRMS analysis. 

#### Chemical characterization

The compound purity and structure were confirmed using the following biophysical techniques. 

#### Fourier Transform Infrared Spectroscopy (FT-IR) 

Thermo Nicolet Nexus 670 Spectrometer was used for FT-IR analysis. Potassium bromide was used as beam splitter, the infra red (IR) with the wavelength of 4000 cm^-1^ to 400 cm^-1^, was used as a radiation source at mid IR region (Majzner et al., 2013[20]). DTGS potassium bromide with resolution of 4 cm^-1^ was used as a detector.

#### Nuclear Magnetic Resonance Spectroscopy (NMR) 

Tetramethylsilane (TMS) was used as the internal standard on a 300, 500 MHz spectrometer for ^1^H NMR and ^13^C NMR spectral recordings respectively. The conditions maintained for recording ^1^H NMR were TMS at 0.00 ppm, CDCl_3_ at 7.26 ppm, where as for ^13^C NMR were CDCl_3_ at 77.0 ppm, DMSO at 39.43 ppm (Morcombe and Zilm, 2003[23]).

#### Electrospray Ionisation Mass Spectrometry (ESI -MS)

Quattro Micro triple quadrupole mass spectrometer (Micromass, Manchester, UK) was used for recording mass spectra (Sridhar et al., 2014[31]). Using autosampler, the samples were introduced into the mass spectrometer with acetonitrile as the mobile phase at 0.2 ml/min. The ESI capillary voltage was maintained at + 4.0 kV, Nitrogen was used as the desolvation gas at 150 °C. The data was acquired using Masslynx software (version 3.2).

#### Electrospray Ionisation High-Resolution Mass Spectrometry (ESI-HRMS)

Exactive Orbitrap mass spectrometer (Thermo Scientific, Waltham, MA, USA) was used to record electrospray ionization high resolution mass spectra, in positive ion mode (Nagaveni and Prabhakar, 2015[24]). Spray voltage was maintained as 4000 V and capillary voltage as 30 V at 250 °C. Using Xcalibur software (Thermo Scientific) data was acquired.

### Biological evaluation 

#### Anti-bacterial activity 

The minimum inhibitory concentrations (MIC) were recorded against *Staphylococcus aureus, Bacillus subtilis, Staphylococcus epidermidis, Pseudomonas aeruginosa, Escherichia coli *and* Klebsiella pneumoniae*. In brief 96-well plates were distributed with Nutrient broth and the compounds in different concentrations (150 μg/mL to 0.5μg/mL). 50 μL microbial suspensions were seeded into each well (0.5 MacFarland standards) to make the final volume of 200 μl with culture broth. The plates were incubated for 24 hrs at 37 °C. The lowest concentration, at which the visible inhibition was recorded, was considered as MIC. Streptomycin and Benzylpenicillin were used as positive controls.

#### Larvicidal bioassay/Dose-response bioassay 

Based on the preliminary screening results, different concentrations ranging from 25 to 200 ppm were prepared for DEHP and 25 to 1 ppm for DBP were used. The compounds were subjected to dose response bioassay against 4^th^ instar larvae of *Aedes aegypti *larvae. Dead larvae were counted after 24 h of exposure, and the percentage mortality was reported from the average of triplicates (Govindarajan et al., 2016[9]). 

#### Acetylcholinestarase activity 

Acetylcholinesterase inhibitory activity was performed according to Ellman's method (Tao et al., 2016[32]) with modifications. The 4^th^ instar larvae of *Aedes aegypti *were homogenised in phosphate buffer (0.1M; pH=7) and centrifuged at 8000 rpm for 30 min at 4 °C. Supernatant was collected and used as enzyme source. Different concentration of the compounds (30 μl) each was added into 96 well microplate along with 30 μl of supernatant and 40 μl of phosphate buffer (0.1 M; pH=8) and incubated for 1 hour. 50 μl each of 0.6 mmol/L DTNB and 1.5 mmol/L acetylthiocholine iodide were added and OD was recorded at 412 nm. Triplicates were maintained. 

#### MTT cell proliferation assay 

The cell lines were seeded in 96-well plates in 200 µL aliquots. The test compounds in different concentrations were added and incubated at 37 °C for 24 hrs. Doxorubicin was used as positive control (Prata-Sena et al., 2016[28]). 

#### Flow cytometry analysis of nuclear DNA 

To analyze the nuclear DNA content (Patel et al., 2016[25]), CHO cells (2 × 10^8^ cells) were treated with pure compounds for 24 hrs at concentrations 150, 175, 200 µg/ml for DEHP and 10, 15, 30 μg/ml for DBP. This is followed by washing the cells twice with ice-cold PBS buffer, harvesting, fixing with cold 70 % ethanol in PBS and storing at −20 °C for 30 min. After fixation, the cells were incubated with Triton-X 100 (50 μg/mL), RNase A (0.1 mg/mL), at 37 °C for 30 min, stained with propidium iodide (50 μg/mL) for 30 min and then measured for nuclear DNA content using Amnis flowsight imaging flow cytometer from M/S Millipore with INSPIRE software.

## Results and Discussion

**Figure 1:**
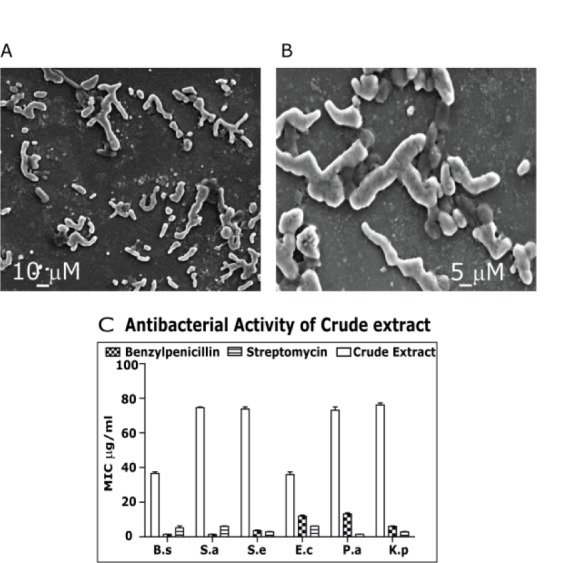
A) Scanning electron micrographs of *Brevibacterium mcbrellneri*: Rod shaped culture was 7-10 µm long and 3-4 µm wide. B) The coccoid shaped culture measures around 5 µm in diameter. C) Anti-bacterial activity of crude extract: MIC values were recorded against representative microorganisms. The antibiotics Benzylpenicillin and Streptomycin were used as positive control*. (B.s = Bacillis subtilis, S.a = Staphylococcus aureus, S.e = Staphylococcus epidermidis, E.c= Escherichia coli, P.a=Pseudomonas aeroginosa, K.p = Klebsiella pneumonia).*

*Brevibacterium mcbrellneri* appears as rod shape in fresh culture and coccoid shape in old culture as described previously (McBride et al., 1993[22]). They are non-spore forming. Both the forms are Gram-positive and were single or sometimes in pairs under light microscope. They appeared as irregular rods or in V shape when observed under the scanning electron microscope (Figure 1A and 1B(Fig. 1)). The bacilli were 5-7 µm long and 3-4 µm wide. The cocci measured around 3 µm in diameter. This is in comparison with *B. massiliense *(Roux and Raoult, 2009[29]). The *B. mcbrellneri* shows Gram positive nature without producing spores that are irregular, short, straight rod measuring 0.4-1.4 mm in length and 0.3-0.5 mm in diameter. 

Crude extract and fractions were checked for anti-bacterial activity to identify the fraction that has to be fractionated further, to isolate the biologically active compounds (Figure 1C(Fig. 1)). After the crude extract was tested for bioactivity, it was subjected to column chromatography with non-polar *n*-hexane and polar ethyl acetate as solvent system. The *n*-hexane fraction of the crude extract furnished a mixture of compounds that were eluted using a polarity gradient of solvent system. The fractionation process was monitored using TLC analysis. The fractions that exhibited similar TLC profiles were pooled and antimicrobial property of the fractions was evaluated. Fraction showing the best activity was again subjected to column chromatography to isolate bioactive molecules. A pale yellow transparent liquid (compound 1), was isolated along with a colourless transparent liquid (compound 2). The chemical structures of the 2 purified compounds were then elucidated on the basis of FTIR, NMR and Mass spectrometry. 

### Compound 1 

**Figure 2:**
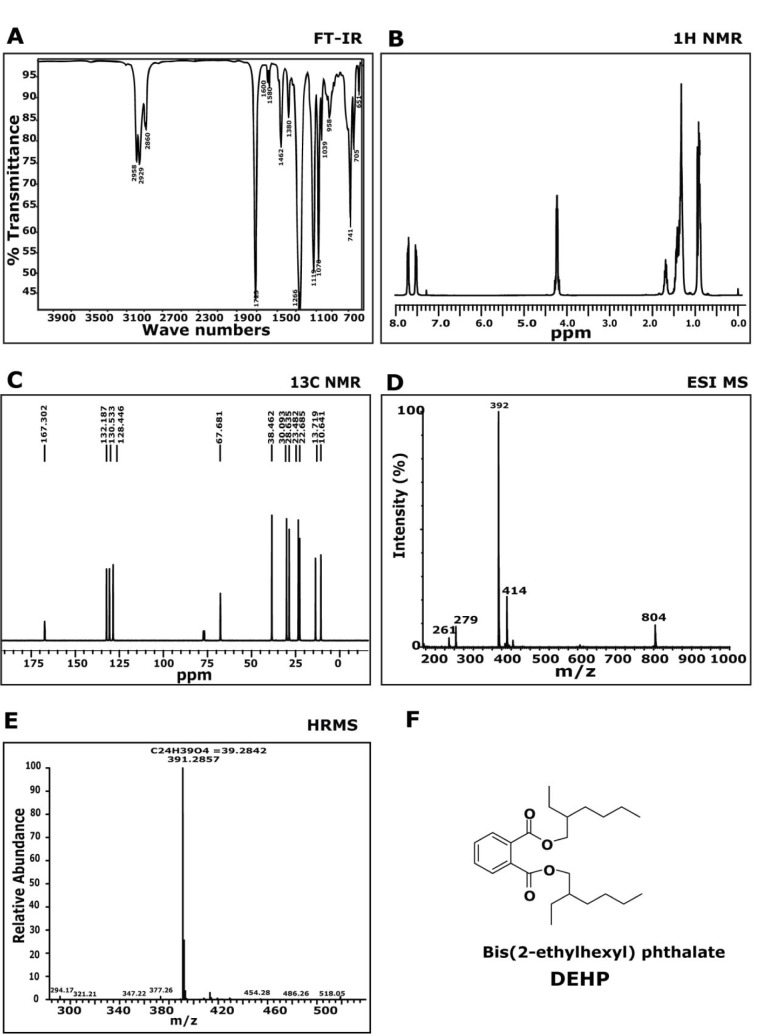
Chemical characterization of Di-(2-ethylhexyl) phthalate: A) FT-IR spectra showed peaks at 2958, 2929, 2860, 1729, 1462, 1380, 1266, 1070, 1039, 741, 651 cm^-1^. B) ^1^H NMR spectra showed peaks at δ 7.72-7.69 (m, 2H), 7.53-7.50 (m, 2H), 4.24-4.21 (m, 4H), 1.45-1.32 (m, 16H), 0.92 (t, 12H). C) ^13^C NMR spectra showed peaks at δ 10.6, 13.7, 22.6, 23.4, 28.6, 130.5, 132.1, 167.3. D) MS(ESI): (m/z) = 391 (M+1)^+^. E) HRMS (ESI) (M+1)^+^ m/z calcd for C_24_H_39_O_4_ = 391.2857, found = 391.2842.

The FTIR spectrometric analysis of compound 1 (Figure 2A(Fig. 2)) showed characteristic absorbance at 1728 cm^-1^, representing carbonyl band and at 1072~1250 cm^-1^, signifying C-O band. This data determines the presence of aromatic ester in the isolated molecule. The aromatic signals between 7.72 (m) and 7.53 (m) ppm, on the ^1^H-NMR spectrum (Figure 2B(Fig. 2)) have coupling constants as predicted for protons at the ortho-substituted ring. Signal at 4.24 (m, 4H) ppm is assigned to a methylene group adjacent to the ester alcohol group. With the available chemical shifts at different peaks, we could determine the possible fragment structure recorded in the spectroscopy.

The ^13^C NMR spectrum (Figure 2C(Fig. 2)), confirmed the symmetry of the molecule, exhibiting the expected 12 carbon resonances (δ 10.6, 13.7, 22.6, 23.4, 28.6, 30.1, 38.4, 67.6, 128.4, 130.5, 132.1, 167.3) assigned as two quaternary, three methane and five methylene carbons with two methyl groups. This data is analogous to ^1^H NMR data and helped in providing the Carbon backbone of the molecule. By comparison of the recorded ^1^H and ^13^C-NMR data to those published in literature (Amade et al., 1994[2]), compound 1 was identified as di-(2-ethylhexyl) phthalate (DEHP). The mass spectrum of the expected molecule was analysed and found to be 391 (M+1)^+ ^(Figure 2D(Fig. 2)). The HRMS was recorded and calculated by using Mass centre software) database (Figure 2E(Fig. 2)), and obtained peak at m/z ratio at 391.2857 was found to match with 391.2842, which corresponds to C_24_H_39_O_4_. Based on FTIR, NMR and MS data the compound was identified as di-(2-ethylhexyl) phthalate (DEHP). 

### Compound 2 

**Figure 3:**
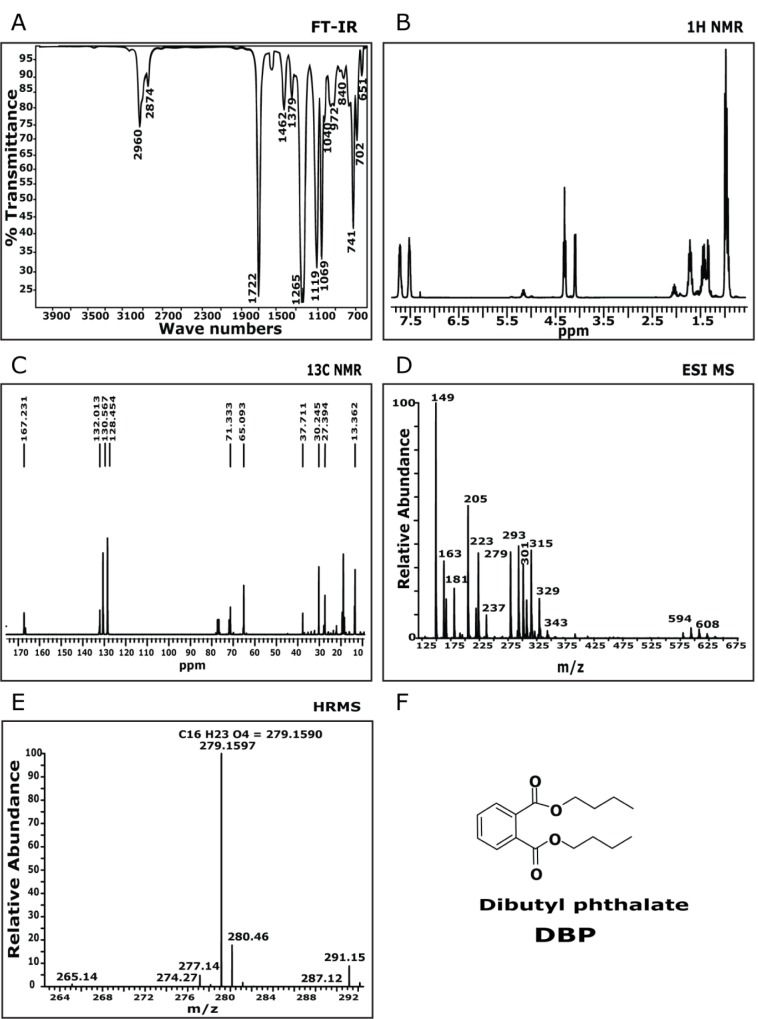
Chemical characterization of dibutyl phthalate: A) FT-IR spectra showed peaks at 2960, 2874, 1722, 1265, 1462, 1265, 1119, 1069, 741, 651 cm^-1^. B) ^1^H NMR spectra showed peaks at δ 7.73-7.70 (m, 2H), 7.53-7.50 (m, 2H), 4.3 (t, 2H), 4.10-4.08 (d, 1H), 2.08-2.02 (m, 1H), 1.47-1.33 (m, 4H), 0.97 (t, 7H). C) ^13^C NMR spectra showed peaks at d 13.3, 18.8, 132.0, 167.2. D) MS(ESI): (m/z) = 279 (M+1)^+^. E) HRMS (ESI) (M+1)^+^ m/z calcd for C_16_H_23_O_4_ = 278.1597, found = 279.1590.

The FTIR spectrum showed a characteristic absorption frequency at 1727 cm^-1^ and this can be attributed to the ester carbonyl group stretching vibration (Figure 3A(Fig. 3)). The band at 1598 cm^-1^ and 1588 cm^-1^ corresponds to the −CH− stretching for aromatic nucleus. A characteristic absorption at 1122 cm^-1^ and 1072 cm^-1^ are attributed to C-O stretching. This data could determine the functional group present in the molecule and could predict that aromatic ester derivative might be present in the isolated molecule. The compound was then subjected to ^1^H NMR (Figure 3B(Fig. 3)) analysis in 500 MHz spectrometer with CDCl_3_ as solvent system which displayed an up field triplet at δ 0.97 for methyl protons. The multiplets at δ 1.47 (4H) and δ 1.74 (3H) represent methylene protons. A downfield triplet at δ 4.3 (4 H), represents methylene protons. The doublet of doublets at δ 7.53 (2 H) and d 7.73 (2H) indicate aromatic protons. This was followed by ^13^C NMR analysis (Figure 3C(Fig. 3)) in 500 MHz spectrometer with CDCl_3_ as solvent system. It displays eight signals accounting for sixteen carbon atoms. Quartet at δ 13.3 is assigned to - 5'methyl carbon atoms. The triplets at δ 18.8, d 30.2 and 65.0 represent methylene carbons. The downfield doublets observed at δ 128.84 and δ 130.5 represent aromatic carbon atoms. A singlet at δ 132.05 indicates tetra substituted aromatic carbon atoms. The most downfield singlet at δ 167.2 is assigned to carbonyl carbon atoms. This data provides the Carbon backbone of the isolated molecule. The mass spectrum (Figure 3D(Fig. 3)) of the expected molecule was analysed and found to be 279 (M+1)^+^. This information provides the molecular mass of the expected molecule. The HRMS (ESI) (Figure 3E(Fig. 3)), was recorded and relative intensity vs. m/z ratio was calculated as 278.1597. This was found to match C_16_H_23_O_4_ = 279.1590. This provides the molecular formula of compound. By comparison of ^1^H and ^13^C NMR data to those published in literature (Roy et al., 2006[30]) and based on FTIR and MS data the isolated compound was identified and confirmed to be dibutyl phthalate.

**Figure 4:**
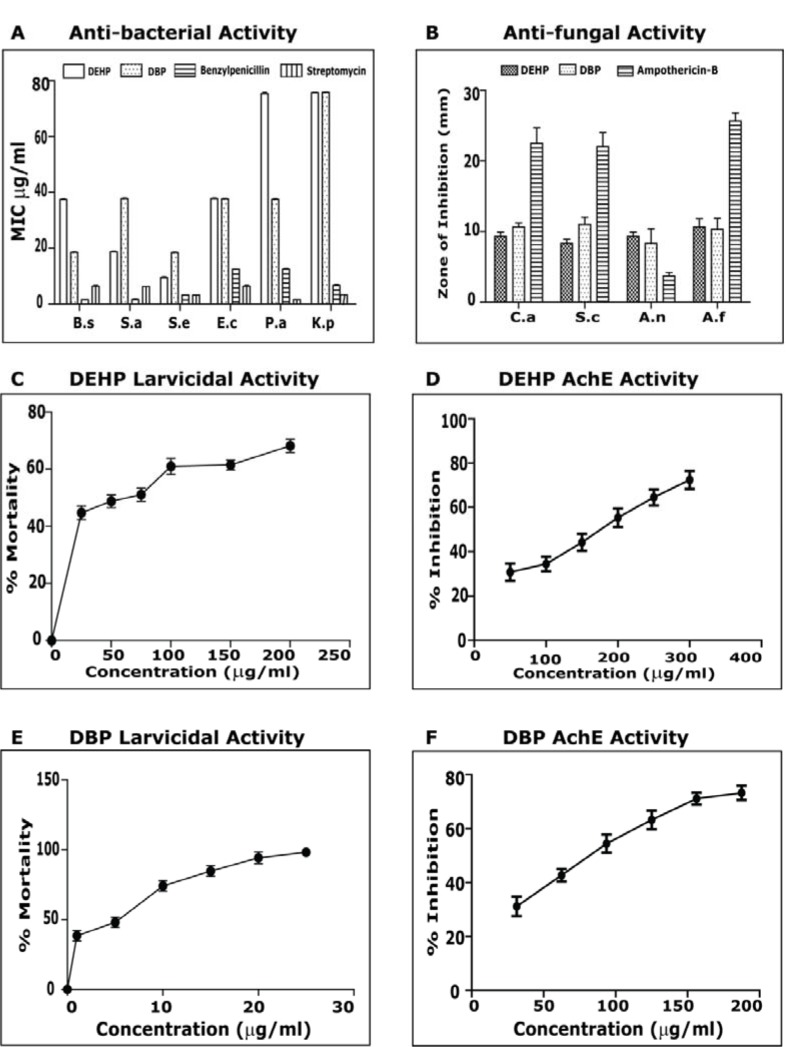
A) Anti-bacterial activity of isolated ester derivatives Di-(2-ethylhexyl) phthalate and Dibutyl phthalate: MIC was calculated. The antibiotics Benzylpenicillin and Streptomycin were used as positive control*. (B.s = Bacillis subtilis, S.a = Staphylococcus aureus, S.e = Staphylococcus epidermidis, E.c= Escherichia coli, P.a=Pseudomonas aeroginosa, K.p = Klebsiella pneumonia)* B) Mosquito larvicidal activity against the Di-(2-ethylhexyl) phthalate. Mortality percentage at particular concentration and 50 percent lethal concentration was calculated. C) Mosquito larvicidal activity against the Dibutyl phthalate. Mortality percentage at particular concentration and 50 percent lethal concentration was calculated. D) Acetylcholinesterase activity against Di-(2-ethylhexyl) phthalate. Percentage inhibition at particular concentration and 50 percent inhibitory concentration was calculated. E) Acetylcholinesterase activity against Dibutyl phthalate. Percentage inhibition at particular concentration and 50 percent inhibitory concentration was calculated.

The compounds were effective against both Gram positive and Gram negative bacteria; the MIC was compared with the standards Penicillin and Streptomycin (Figure 4A(Fig. 4)). Anti-bacterial activity of isolated ester derivatives di-(2-ethylhexyl) phthalate and dibutyl phthalate). DEHP was most effective against *S. epidermidis *with MIC of 9.37 µg/ml followed by *S. aureus *(18.75 µg/ml). It was equally effective against *B. subtilis *along with* E. coli *showing an MIC of 37.5 µg/ml. It showed activity against *P. aeroginosa *and* K. pneumoniae *at 75 µg/ml. DEHP's effect against *S. epidermidis *was comparably close to the standard antibiotics. DBP had maximum effect on both *B. subtilis* and *S. epidermidis* showing activity at 18.75 µg/ml. It had equal activity against *E. coli, P. aeroginosa *and* S. aureus *(MIC= 37.5 µg/ml). It showed activity against *K. pneumoniae *at 75 µg/ml.

Previously there are reports of antimicrobial activity on DEHP isolated from *Calotropis gigantean* showing MIC at 32 µg/ml against *B. subtilis* and *Sarcina lutea* and with negligible antifungal property (Habib and Karim, 2009[10]). There are also reports of antimicrobial activity on DBP isolated from *Streptomyces albidoflavus* showing MIC on *E. coli* 53 µg/ml, *B. subtilis* 84 µg/ml, *S. typhi* 76 µg/ml, *S. cerevisiae* 92 µg/ml, *A. niger *98 µg/ml, *Curvularia pallescens* 117 µg/ml (Roy et al., 2006[30]). DEHP and DBP were also evaluated for their toxicity against the larvae of *Ae. aegypti.* The compounds caused mortality in 4^th^ instar of *Aedes aegypti* after 24 hrs (Figure 4B and 4C(Fig. 4)). Mosquito larvicidal activity against the di-(2-ethylhexyl) phthalate and dibutyl phthalate). The highest mortality was observed in DBP, with LC_50_ at 1.62 ppm. It caused 100 % mortality at 25 ppm. DEHP showed LC_50_ at 47.65 ppm. The effect of different concentrations, ranging from 50-300 µg/ml was observed on AChE enzyme activity, and the % inhibition was recorded (Figure 4D and 4E(Fig. 4)). Acetylcholinesterase activity against di-(2-ethylhexyl) phthalate and dibutyl phthalate). For DHEP and DBP the IC_50_ was recorded as 173.29±4.8 µg/ml and 138.92±6.6 µg/ml respectively. This is the first report on DEHP and DBP showing AChE inhibition, against mosquito larva* Aedes aegypti*. Previously there are reports of these compounds showing AChE inhibition, against embryonic Zebra fish (Xu et al., 2013[35]). 

**Figure 5:**
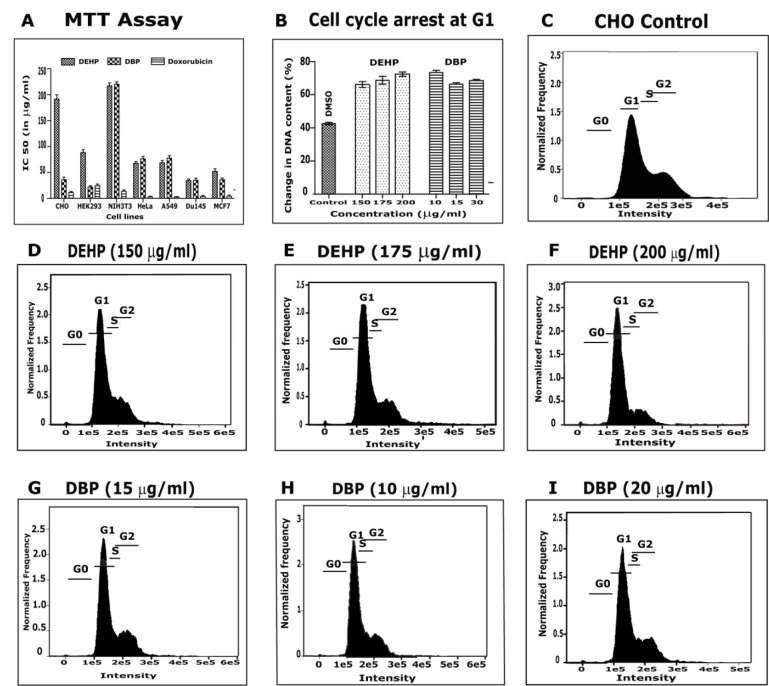
Cytotoxicity Assessment: A) MTT proliferation assay. Doxorubicin used as positive control. B) Analysis of DNA content in CHO cells treated with DEHP and DBP in comparison to control. DMSO used as negative control. C) Cell cycle analysis in CHO cells with DMSO. (D-F) Cell cycle analysis in CHO cells with different concentrations of DEHP. (G-I) Cell cycle analysis in CHO cells with different concentrations of DBP.

The effect of DHEP and DBP on the proliferation of normal and cancer cell lines, with doxorubicin as positive control, was evaluated by MTT cell proliferation assay (Figure 5A(Fig. 5)). DBP showed cytotoxicity against CHO with IC_50_ = 36±8.6 µg/ml whereas DEHP could show mild cytotoxicity with IC_50_ = 193±5.4 µg/ml. DBP was also effective against HEK 293 with IC_50_ = 19.77±10 µg/ml which is more toxic than the standard doxorubicin with IC_50_ = 21.80±8.8 µg/ml. DHEP and DBP showed IC_50_ = 33.46±7.7 µg/ml and 31.23±8.7 µg/ml respectively, against DU-145. DHEP and DBP were active against MCF-7 with IC_50_ = 46.55±0.1 µg/ml and 32.43±3.6 µg/ml respectively. From the above study we could say that both DHEP and DBP showed cytotoxicity properties in normal cells and also to all the cancer cell lines in a dose-dependent manner. Cytotoxic activity of DBP has been reported against A549 cells (Hsu et al., 2011[14]). Based on the MTT cell proliferation assay, we carried out FACS studies to verify the toxic effect of isolated compounds on cell cycle and DNA content in CHO cell line (Figure 5B(Fig. 5)). In both the compounds tested concentrations revealed significant inhibition of cell proliferation at the G1/S boundary leading to an increased accumulation of cell population in G1 phase of the cell cycle, with relative significant depletion of cell number in S phase in CHO cells. At the G1 gate, cell population was noticed to increase from 20 % to 28 % with a corresponding decrease of 21 %-22 % of cells in S-phase indicating that both the compounds at all the exposed concentrations caused arrest of cells at the G1 checkpoint and inhibited further DNA synthesis of cells. FACS analysis (Figure 5(Fig. 5)) demonstrated the presence of 46 % of cells in G1 phase; 25.3 % in S-phase, 26.4 % in G2/M and 1.9 % of cells in SubG_0_/G_1_ with DMSO after 24 hr exposure. Previously there are reports on DEHP showing cell cycle arrest at G1 phase of growth in HaCaT cells (Peropadre et al., 2015[26]) and that DEHP could show apoptosis without cell cycle arrest HePG2 cells (Lyu et al., 2016[19]). 

## Conclusion

In our present study two phthalate derivatives DEHP and DBP were isolated from *Brevibacterium mcbrellneri*. From our toxicological and biochemical evaluation studies we infer that these compounds have antibacterial activity and mosquito larvicidal activity with significant acetylcholinesterase inhibition. These phthalate derivatives have also shown cytotoxicity against all cell lines tested by showing cell cycle arrest in G1 phase. 

## Notes

Ramars Amanchy and Suryanarayana Murty Upadhyayula (Biology Division, CSIR-Indian Institute of Chemical Technology (IICT), Uppal Road, Tarnaka, Hyderabad -500 007, India; Fax: +91-040-27193227, Tel: (+) 91-40-27193134, E-mail: ramars@iict.res.in, director@niperguwahati.ac.in) contributed equally as corresponding authors.

## Acknowledgements

We would like to thank Department of Science and Technology, India, for the DST Inspire fellowship (GAP-0347).

## Conflict of interest

The authors would like to declare no conflict of interest.
